# Multicenter Untargeted Metabolomics Study of Dementia: Investigating Potential Biomarkers and Pathways

**DOI:** 10.1002/alz70856_102823

**Published:** 2025-12-24

**Authors:** Mai Othman, Mohamed Salama

**Affiliations:** ^1^ Institute of Global Health and Human Ecology, School of Science and Engineering, The American University in Cairo, Cairo, New Cairo, Egypt; ^2^ Institute of Global Health and Human Ecology at the American University in Cairo, Cairo, Cairo, Egypt

## Abstract

**Background:**

Dementia represents a significant global health challenge, with its prevalence steadily increasing; however, research on its metabolomic dimensions remains scarce, particularly within underrepresented populations. Gaining a deeper understanding of the metabolic changes underlying the disease is essential for advancing both diagnostic and therapeutic approaches. This exploratory untargeted Liquid Chromatography‐Mass Spectrometry (LC‐MS) metabolomic multi‐center study seeks to elucidate the metabolic alterations linked to dementia within an Egyptian population, offering valuable insights into potential biomarkers and pathways that may be unique to this region.

**Method:**

Serum samples were collected from 150 dementia patients and 100 age‐ and sex‐matched controls from multiple centers spanning Egypt. These samples were processed and analyzed using LC‐ESI‐MS/MS with the ExionLC AC‐SCIEX Triple Quad 5500+ MS/MS system (Sciex) employing both positive and negative ionization modes to capture a wider range of metabolites. Untargeted metabolomics experiments were performed using Data‐Dependent Acquisition (DDA) mode. Comprehensive data acquisition, peak identification, and quality control procedures were applied. Statistical analyses were conducted to determine differentially expressed metabolites, and pathway enrichment analysis was performed to assess the biological relevance of the results.

**Result:**

A total of 253 metabolites were identified and categorized into lipids, amino acids, nucleosides, and small molecules. Principal Component Analysis (PCA) revealed distinct clustering patterns in the metabolomic profiles of dementia patients when compared to controls. Regression analysis model demonstrated significant differential abundance of various metabolite groups between the two cohorts. Pathway enrichment analysis further highlighted disruptions in pathways related to metabolism, lipid homeostasis, signaling, protein synthesis, and inflammation, underscoring the complex interactions between metabolic processes and the pathogenesis of dementia.

**Conclusion:**

This exploratory untargeted LC‐MS metabolomic study offers valuable insights into the metabolic landscape of dementia patients within the previously underexplored Egyptian population. The metabolites and pathways identified in this study contribute to the broader understanding of dementia and provide potential biomarkers and metabolic pathways associated with the disease. Further validation focusing on specific metabolites is necessary to confirm these preliminary findings and support the development of targeted interventions for dementia in this population.

